# 

**Published:** 1895-04-20

**Authors:** 


**Notices of Births, Deaths, and Marriages** are inserted in this column at a charge of 2s. 6d. for an announcement not exceeding four lines.

**Notice to Advertisers and Subscribers.**

All Advertisements, Orders for Copies of The Hospital and Business Communications should be addressed to **The Manager, NOT TO THE EDITOR,** The Hospital, 428, Strand, London, W.C.

The Cost of Subscription, post free, is as follows:—Per week, 2½d.; per quarter, 2s. 8d.; per half-year, 5s. 3d.; per annum, 10s. 6d. Foreign:—Per Annum, 15s. 2d.; payable, in all cases, in advance.

**NOTICE TO CORRESPONDENTS.**

All MS., Letters, Books for Review, and other matters intended for the Editor should be addressed The Editor, The Lodge, Porchester Square, London, W.

The Editor cannot undertake to return rejected MS., even when accompanied by stamped directed envelope.

**"Burdett's Hospital Annual," 1895,**

Ready Immediately.

Sixth year of publication. About 1000 pp. Scarlet cloth, gilt lettered. Price 5s. A most complete guide to British, Colonial, Indian, and American Hospitals, Dispensaries, Nursing and Convalescent Institutions, and Asylums. A few copies of the "Annual" for 1894 may still be obtained on application to the Publishers, The Scientific Press (Limited), 428, Strand, London, W.C.

**NOTICE.—The Index for Vol. XVII. (ending March 30th, 1895) is now on sale at the Office of this Journal. Price 2½d., post free.**

### The Student of Medicine.

### Appointments.

G. S. Buchanan, M.D., B.S., B.Sc.Lond., appointed Medical Inspector of the Local Government Board. F. F. Burghard, M.S.Lond., F.R.C.S., appointed Surgeon to Out Patients at Paddington Green Children's Hospital. F. H. Cooke, M.R.C.S., L.R.C.P.Lond., appointed Medical Officer for the Birch District of the Lexden and Winstree Union. W. T. Davies, appointed Medical Officer for the Western District of the Llandilofawr Union. E. P. Dickin, M.B., C.M.Edin., appointed Assistant House Surgeon to the General Infirmary, Northampton. J. D. Dickson, M.D.Q.U.I., L.R.C.S.I., appointed Medical Officer of Health for the Wycombe District. C. R. Edmondson, M.B., C.M.Edin., appointed Senior House Surgeon to the Royal Southern Hospital, Liverpool. C. E. S. Flemming, M.R.C.S.Eng., appointed Medical Officer for the No. 8 District of the Bath Union. L. Gilbes, M.R.C.S., L.R.C.P.Lond., appointed Second Assistant Medical Officer to the St. Marylebone Infirmary, Notting Hill. H. Hollis, B.A., M.B., B.C.Cantab., appointed House Surgeon to the General Infirmary, Northampton. G. H. Lock, M.R.C.S., L.R.C.P., appointed House Surgeon to Paddington Green Children's Hospital. T. Macdonald, M.B., C.M.Edin., appointed Medical Officer for the Parish of Kiltarlity. T. F. McDonald, M.B., appointed Medical Officer for the Workhouse and the Hellingly District of the Hailsham Union. J. Marsh, M.B., C.M.Edin., appointed Second House Surgeon to the Royal Southern Hospital, Liverpool. J. Paterson, appointed Medical Officer for the Berriew District of the Forden Union. J. Poland, F.R.C.S.Eng., appointed Surgeon to the City Orthopædic Hospital. R. D. Prichard, L.R.C.P., L.R.C.S.Edin., L.F.P.S.Glasg., appointed Medical Officer for the Second Central District of the Neath Union. J. A. Sanderson, L.R.C.P. and S.Edin., appointed Junior House Surgeon to the Royal Southern Hospital, Liverpool. U. R. I. Smythe, M.B.Lond., appointed House Physician to the Workhouse Infirmary, Birmingham. Dr. Thompson, appointed Medical Officer to the Launceston Post Office. W. S. West, M.A., M.B., B.C.Cantab., appointed Obstetric House Physician to the Middlesex Hospital.

### Vacancies.

The following vacancies are announced this week, the amount of salary in each case being given after the name of the institution:

**Resident Medical Officers.**

Birmingham General Dispensary.—Resident Surgeon; doubly qualified. Salary £150 per annum (with an allowance of £30 per annum for cab hire), and furnished rooms, fire, lights, and attendance. Applications to Alex. Forrest, Secretary, by April 22nd.

Bradford Infirmary and Dispensary.—Junior House Surgeon; unmarried. Salary £50 per annum, with board and residence. Applications, endorsed "Junior House Surgeon," to William Maw, Secretary, by April 23rd.

Hospital for Sick Children, Great Ormond Street, Bloomsbury, W.C.—Resident House Physician; also House Surgeon. Appointment for six months. Salaries £20, with board and residence in the hospital. Unmarried, and possess a legal qualification to practice. Applications and testimonials to Adrian Hope, Secretary, before Tuesday, April 30th.

Kent County Lunatic Asylum, Barming Heath, near Maidstone.—Fourth Assistant Medical Officer and Pathologist; unmarried. Salary £175 per annum, rising £5 annually, with furnished quarters, attendance, coal, gas, produce, milk, and washing. Appointment for two years in the first instance. Applications to Francis R. Howlett, Clerk to the Sub-committee of Visitors, 9, King Street, Maidstone, by May 1st.

North-West London Hospital, Kentish Town Road, N.W.—Resident Medical Officer and Assistant Resident Medical Officer. Appointment for six months. Salary at the rate of £50 per annum is attached to the senior post. Applications to the Secretary by April 26th.

Royal Hospital for Diseases of the Chest, City Road, E.C.—Resident Medical Officer. Appointment for six months. Salary £100 per annum, with furnished apartments and board. Applications to the Secretary by April 24th.

Royal South Hants Infirmary, Southampton.—Assistant House Surgeon; must be willing to engage for six months. Gratuity of £10 will be given at the end of this period if satisfactory. Board and lodging provided. Applications to T. A. Fisher Hall, Secretary, by April 24th.

St. Luke's Hospital, E.C.—Clinical Assistant; must be duly qualified to practise and registered. Appointment for six months, with board and residence. Applications and testimonials to Percy de Bathe, M.A., Secretary, by April 22nd.

Tancred's Charities.—One Studentship in Physic at Gonville and Caius College, Cambridge. The student will receive £50 a year and a share in the surplus rents and profits (the stipend not to exceed £100 a year) until admission to the degree of Bachelor of Medicine, and for three years afterwards, or for such shorter terms as may be determined by authority. Candidates must not be below the age of 16 or above the age of 22. Forms of petition and all information may be obtained from Mr. George Edgar Frère, 28, Lincoln's Inn Fields, W.C., Clerk to the Tancred's Charities, to whom petitions must be sent by April 20th.

Weston-super-Mare Hospital and Dispensary.—Medical Officer to the Provident Dispensary; must possess a recognised medical and surgical qualification; must be registered under the Medical Act. Salary £80 per annum, with board, lodging, and washing. Applications and testimonials to the Honorary Secretary before April 30th.

**Non-Resident Medical Officers.**

British Lying-In Hospital, Endell Street, Long Acre, E.C.—Physician to the Out-patient Department. Applications to the Secretary by April 20th.

**THE STUDENT OF MEDICINE—*continued.***

Hospital for Sick Children, Great Ormond Street, W.C.—Surgical Registrar and an Anæsthetist. Appointments for one year. Honorariums of £40 and £15 15s. respectively at the expiration of term. Applications to the Secretary by April 23rd.

London Hospital Medical College.—Assistant Demonstrator in Anatomy. Salary £90 per annum. Applications to the Warden by April 25th.

Middlesex Hospital, W.—Assistant Surgeon; must be Fellow (or have passed the qualifying examination for the Fellowship) of the Royal College of Surgeons of England. Applications to F. Clare Melhado, Secretary Superintendent, by May 1st.

Paddington Green Children's Hospital, W.—Surgeon to the Throat and Ear Department. Applications to the Secretary by April 22nd.

Poplar Hospital for Accidents, Blackwall, E.—Honorary Surgeon. Applications to the House Governor by April 20th.

Royal College of Surgeons of England.—Four Examiners in Anatomy and four Examiners in Physiology for the Fellowship; candidates for the former must be Fellows of the College. Four Examiners in Elementary Anatomy (First Examination). Two Examiners in Elementary Biology (First Examination). Four Examiners in Anatomy (Second Examination). Three Examiners in Physiology (Second Examination). Four Examiners in Midwifery (Third Examination). Two Examiners in Public Health. Applications to the Secretary by April 25th.

University of Glasgow.—Two Examiners for Degrees in Medicine to examine in Clinical Medicine and Clinical Surgery respectively. Appointment to last till December 31st, 1898, at the rate of £50 annually. Applications to Alan E. Clapperton, Secretary of the Glasgow University Court, 91, West Regent Street, Glasgow, by May 1st.

### Medical and Administrative Appointments.

### THE HOSPITAL FOR SICK CHILDREN, GREAT ORMOND STREET, BLOOMSBURY, W.C.

ADRIAN HOPE, Secretary.

9th April, 1895. (259)

